# Knockdown of lncRNA TP53TG1 Enhances the Efficacy of Sorafenib in Human Hepatocellular Carcinoma Cells

**DOI:** 10.3390/ncrna8040061

**Published:** 2022-08-10

**Authors:** Qingchun Lu, Mingyang Xin, Qian Guo, Brad S. Rothberg, Ana M. Gamero, Ling Yang

**Affiliations:** Department of Medical Genetics and Molecular Biochemistry, Lewis Katz School of Medicine at Temple University, Philadelphia, PA 19140, USA

**Keywords:** long non-coding RNA, TP53TG1, sorafenib, hepatocellular carcinoma, efficacy

## Abstract

The multikinase inhibitor, sorafenib, is a first-line treatment for hepatocellular carcinoma (HCC), but its limited efficacy, drug resistance and toxicity are a concern. In this study, we investigated the role of lncRNA TP53TG1 in the efficacy of sorafenib in HCC cells. We found that treatment with sorafenib increased the expression of TP53TG1 in HCC cells. Knockdown of TP53TG1 sensitized tumor cells to the antiproliferative effects of sorafenib. Furthermore, TP53TG1 knockdown had an additive inhibitory effect on HCC cell proliferation and migration in the presence of sorafenib. The combination of TP53TG1 knockdown and sorafenib drastically inhibited the activation of the ERK pathway. This work demonstrates that TP53TG1 deficiency enhances the efficacy of sorafenib in HCC. Combining TP53TG1 knockdown with sorafenib may be an optimal form of therapy for HCC treatment.

## 1. Introduction

Hepatocellular carcinoma (HCC) is the fourth most common cause of cancer-related death worldwide, with a low five-year survival rate of 18.1% [1]. For advanced HCC, sorafenib is the global gold standard and first-line therapy, as neither surgery nor loco-regional curative treatment is suitable [2]. However, more than 30% of patients who received sorafenib suffer from severe adverse events, such as hypertension, bleeding, and cardiac events [3,4]. Sorafenib-associated toxicities lead to dose reduction or discontinuation of treatment [5]. Decreasing the dose of sorafenib while maximizing the treatment outcome is a promising strategy for minimizing adverse events. Combination therapies have been demonstrated to reduce the dosage requirement for each individual agent and maximize the overall efficacy [6]. Therefore, an approach that combines sorafenib with other reagents to improve the efficacy of sorafenib in HCC is urgently needed.

Sorafenib is a multikinase inhibitor that targets the RAF serine/threonine kinases of the RAS-RAF-MEK-ERK (ERK cascade) signaling pathway [7]. Activation of the ERK cascade occurs in most cancer types including HCC [8]. Blocking ERK activity suppresses cell proliferation, migration, and invasion of tumors [9]. More importantly, some ERK cascade inhibitors have been approved for clinical use to treat cancers [10]. For example, the BRAF inhibitors, encorafenib and binimetinib, have been approved for the treatment of metastatic melanoma, whereas sorafenib is approved to treat kidney cancer [11,12]. Therefore, the ERK signaling pathway is considered a major target for developing therapeutic approaches against various cancers including HCC.

Long non-coding RNAs (lncRNAs) are a group of transcripts without protein-coding ability [13]. LncRNAs play important roles in cancer development and are attractive targets for cancer treatment [14]. It has been reported that lncRNAs can manipulate the efficacy of sorafenib in cancer treatment via different mechanisms [15]. For example, the depletion of lncRNA SNHG1 potentiated the efficacy of sorafenib by suppressing the activation of Akt signaling in HCC [16]. Another lncRNA, NEAT1, contributed to sorafenib efficacy by interacting with miR-204/ATG3 to promote autophagy in HCC [17]. These data suggest that lncRNAs are important modulators in sorafenib efficacy in cancer cells. A deeper understanding of the roles of lncRNAs in sorafenib sensitivity may lead to novel approaches to improve sorafenib efficacy.

TP53TG1 has been demonstrated to be an important regulator in a series of diseases. For example, TP53TG1 promotes the development of pancreatic ductal adenocarcinoma and cervical cancer via interacting with miR-96 and miR-33a-5p, respectively [18,19]. Another study showed that TP53TG1 plays an anti-fibrotic role to attenuate idiopathic pulmonary fibrosis [20]. However, the role of TP53TG1 in sorafenib efficacy has never been reported before. In our previous study, we reported that lncRNA TP53TG1 functions as an oncogene in HCC [21]. Specifically, knockdown of TP53TG1 suppresses HCC cell proliferation and migration by inhibiting ERK activation. In this study, we found that TP53TG1 expression is increased by sorafenib treatment in HCC cells. The combination of TP53TG1 siRNA and sorafenib treatment blocks HCC cell proliferation and migration via inhibiting ERK signaling. The additive effect of sorafenib and TP53TG1 siRNA on HCC cells suggests that TP53TG1 is an attractive target for enhancing the efficacy of sorafenib therapy.

## 2. Results

### 2.1. Sorafenib Induces lncRNA TP53TG1 Expression

**Figure 1:**
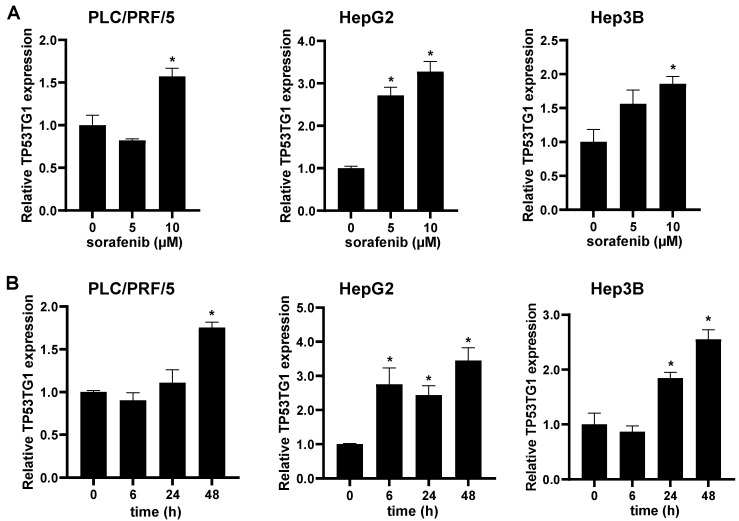
TP53TG1 is upregulated by sorafenib in HCC cells. (**A**) RNA expression levels of TP53TG1 in PLC/PRF/5, HepG2, and Hep3B cells treated with 0, 5, or 10 µM of sorafenib for 48 h (*n* = 3); (**B**) RNA expression levels of TP53TG1 in PLC/PRF/5, HepG2, and Hep3B cells treated with 10 µM sorafenib for 0, 6, 24, and 48 h (*n* = 3). Error bars are shown as standard error of the mean (SEM), * *p* < 0.05.

To determine whether the expression of TP53TG1 is induced by sorafenib treatment, the HCC cell lines HepG2, PLC/PRF/5, and Hep3B were incubated with various concentrations of sorafenib (0, 5, and 10 µM) for 48 h (Figure 1A). We found that the expression level of TP53TG1 was significantly upregulated in a dose-dependent manner. Next, we performed a time-course analysis with 10 µM sorafenib (0, 6, 24, and 48 h) and found that TP53TG1 expression was induced in a time-dependent manner (Figure 1B). This finding suggests that the sorafenib upregulation of TP53TG1 may play a role in the therapeutic efficacy of sorafenib in HCC.

### 2.2. TP53TG1 Knockdown Increases Sorafenib Sensitivity

**Figure 2:**
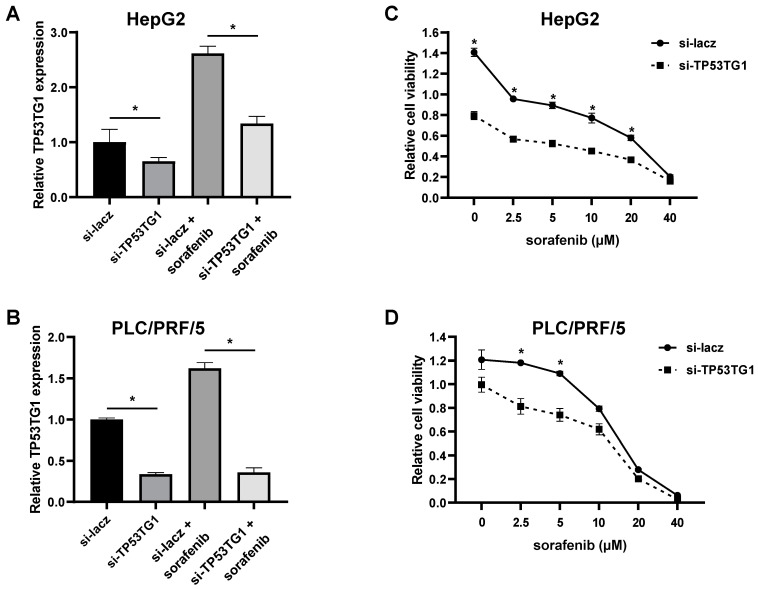
Knockdown of TP53TG1 enhances sensitivity of HCC cells to sorafenib. (**A**,**B**) RNA expression levels of TP53TG1 (normalized to 18S) in (**A**) HepG2 and (**B**) PLC/PRF/5 cells (*n* = 3); (**C**,**D**) Cell viability of (**C**) HepG2 and (**D**) PLC/PRF/5 cells treated with different doses of sorafenib for 48 h (*n* = 6). Error bars are shown as SEM, * *p* < 0.05.

Given that TP53TG1 expression increased upon sorafenib treatment, we investigated the effect of TP53TG1 knockdown on sorafenib efficacy in HCC cells. Since the HepG2 and PLC/PRF/5 showed the highest and lowest expression levels of TP53TG1 upon sorafenib treatment, respectively, they were chosen for the following experiments. We first determined the knockdown efficiency of TP53TG1 in HepG2 and PLC/PRF/5 cells transfected with either control (si-lacz) or TP53TG1 siRNA (si-TP53TG1). About 50–80% of TP53TG1 expression was reduced in TP53TG1 knockdown cells treated with or without sorafenib (Figure 2A,B). Next, we conducted a cell proliferation assay to determine the effect of TP53TG1 knockdown on sorafenib sensitivity. Twenty-four hours after transfection, tumor cells were treated with increasing concentrations of sorafenib (0, 2.5, 5, 10, 20, and 40 µM) for another 48 h. We found that TP53TG1 knockdown sensitized cells to the antiproliferative effects of sorafenib in a dose-dependent manner (Figure 2C,D). These results suggest that TP53TG1 increases the sensitivity of HCC cells to sorafenib.

### 2.3. TP53TG1 Knockdown Has a Synergetic Effect with Sorafenib on Cell Proliferation and Migration

**Figure 3:**
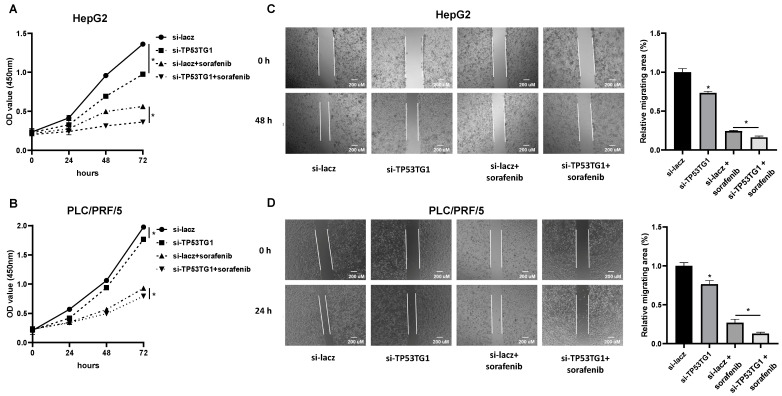
Knockdown of TP53TG1 improves the effect of sorafenib on HCC cell proliferation and migration. (**A**,**B**) Proliferation rate of (**A**) HepG2 and (**B**) PLC/PRF/5 cells treated with si-lacz, si-TP53TG1, si-lacz + sorafenib, and si-TP53TG1 + sorafenib (*n* = 6); (**C**,**D**) Cell migration rate in (**C**) HepG2 and (**D**) PLC/PRF/5 cells treated with si-lacz, si-TP53TG1, si-lacz + sorafenib, and si-TP53TG1 + sorafenib. Error bars are shown as SEM, * *p* < 0.05.

ERK signaling plays a central role in cell proliferation and migration [9]. Our previous study demonstrated that TP53TG1 regulates HCC cell proliferation and migration through ERK signaling [21]. Considering that both sorafenib and TP53TG1 regulate ERK signaling, we next evaluated the combined effect of sorafenib and TP53TG1 knockdown on cell proliferation and migration. For cell proliferation, HepG2 and PLC/PRF/5 cells were transfected with siRNAs, and 24 h later they were treated with either vehicle or sorafenib for different times (0, 24, 48, and 72 h) (Figure 3A,B). The migration rate was determined at 24 h for PLC/PRF/5 or 48 h for HepG2 after treatment with vehicle or sorafenib (Figure 3C,D). The results showed that either TP53TG1 siRNA or sorafenib alone decreased the proliferation and migration rate of cells. More importantly, TP53TG1 siRNA enhanced the anti-cancer effects of sorafenib (Figure 3A–D). Taken together, these results suggest that knockdown of TP53TG1 improves the efficacy of sorafenib in HCC cells.

### 2.4. TP53TG1 May Affect Sorafenib Efficacy through ERK Signaling

**Figure 4:**
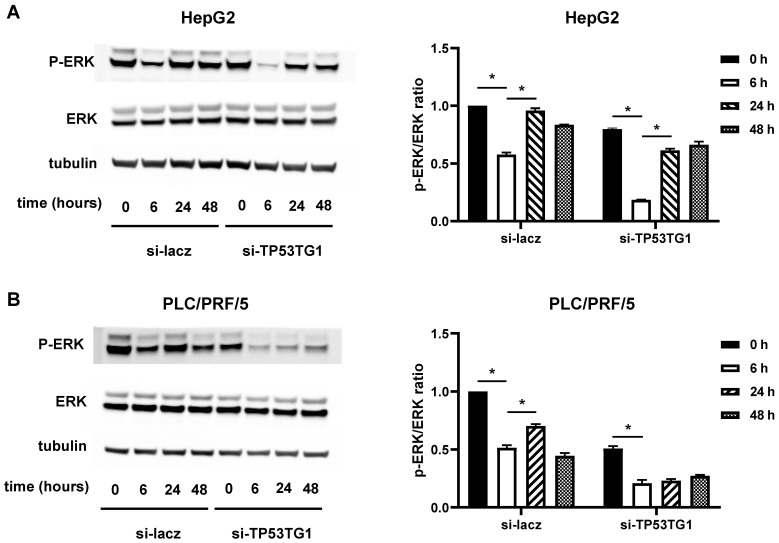
Knockdown of TP53TG1 increases inhibition of ERK activation by sorafenib. (**A**,**B**) Detection of p-ERK and ERK protein content in (**A**) HepG2 cells and (**B**) PLC/PRF/5 cells treated with 10 µM sorafenib at different time points using western blot. Quantification is shown in the right panel (*n* = 3). Error bars are shown as SEM, * *p* < 0.05.

Sorafenib inhibits HCC progression by targeting the ERK signaling pathway [22]. In our previous study, we found that TP53TG1 functions by activating ERK signaling activation in HCC [21]. Therefore, we investigated whether TP53TG1 diminished the sorafenib efficacy in HCC via the activation of ERK signaling. We transfected HepG2 and PLC/PRF/5 cells with si-lacz or si-TP53TG1. Twenty-four hours later, we performed a time course (0, 6, 24, and 48 h) with cells incubated with 10 µM sorafenib. The levels of phosphorylated ERK (p-ERK) were measured by western blot analysis. In the si-lacz group, the p-ERK levels decreased after 6 h of sorafenib treatment and were fully or partially restored at 24 h. In the TP53TG1 knockdown group, however, the p-ERK levels decreased further upon sorafenib treatment compared to the si-lacz group at each time point (Figure 4A,B). These results indicate that TP53TG1 deficiency may improve the therapeutic efficacy of sorafenib via the inhibition of ERK signaling.

## 3. Discussion

LncRNAs have been demonstrated to regulate the chemotherapeutic efficacy of sorafenib in HCC. In this study, we found that sorafenib induced lncRNA TP53TG1. Knockdown of TP53TG1 plus sorafenib treatment had an additive inhibitory effect on the proliferation and migration of HCC cells. Taken together, our findings suggest that TP53TG1 deficiency sensitizes HCC to the antitumor effects of sorafenib.

Several studies indicated that TP53TG1 increases the efficacy of anti-cancer therapy in multiple types of cancer. In non-small cell lung cancer, TP53TG1 enhances the anti-cancer effect of cisplatin by working as a ceRNA of miR-18a [23]. Another study showed that TP53TG1 contributes to the radioresistance of glioma cells by interacting with miR-524-5p [24]. A different study, however, showed that TP53TG1 is a tumor suppressor by inhibiting the activation of the PI3K/Akt pathway to enhance the sensitivity of colon cancer cells to PI3K inhibitor KU-55933 and AKT inhibitor perifosine [25]. In this study, we found that the induction of TP53TG1 by sorafenib may be partially responsible for reducing the therapeutic efficacy of sorafenib. Additionally, Figure 2C,D show a great reduction of cell viability (approximately 40–50%) with TP53TG1 silencing or low doses of sorafenib in HepG2 cells. In contrast, PLC/PRF/5 cells need combination treatment to reach a similar reduction of cell viability for low doses of sorafenib. These results indicate that the role of TP53TG1 in anti-cancer therapy resistance is highly cell specific. Combined with our previous findings that TP53TG1 is an oncogene in HCC, the role of TP53TG1 in sorafenib treatment further suggests the importance of TP53TG1 in HCC.

Sorafenib is a multikinase inhibitor that suppresses cancer progression by mainly inhibiting the RAF-MEK-ERK cascade [26]. However, a previous study reported that sorafenib treatment could induce a rapid increase of p-ERK in BRAF-wild type HCC cell lines due to the dimerization of RAF [27]. This paradoxical activation of ERK signaling limits the therapeutic efficacy of sorafenib in HCC [28]. This phenomenon is consistent with our finding that sorafenib treatment decreased p-ERK at 6 h but then returned at 24 h. We also found that knockdown of lncRNA TP53TG1 significantly decreased the upregulation of p-ERK by sorafenib at 24 h. These results reveal that TP53TG1 inhibition may avoid the paradoxical activation of ERK signaling, thus improving the efficacy of sorafenib in HCC. However, the mechanism by which TP53TG1 regulates ERK signaling remains unknown.

Because the role and mechanism of lncRNAs are complex and highly cell- and tissue-specific, it is difficult to predict the detailed mechanism by which TP53TG1 affects p-ERK in HCC. One possibility is that TP53TG1 might affect p-ERK by sponging miR-219a-2-3p, a microRNA known to regulate p-ERK activation and an interacting microRNA with TP53TG1 in the ENCORI database. Another potential explanation is that TP53TG1 affects p-ERK via interaction with other miRNAs, such as miR-33b or miR-524-5p, which are known regulators of p-ERK [24,29,30,31]. Besides working as a miRNA “sponge”, TP53TG1 can also bind to proteins directly to affect tumorigenesis [25]. It would be interesting to investigate the detailed mechanism in the future. In addition, the TP53TG1 knockout HCC cell line, sorafenib-resistance HCC cell line, and normal hepatocyte cell line can be considered in future studies to further determine the therapeutic effect and mechanism of TP53TG1 in HCC.

The siRNA-based therapeutic approach is an effective and promising method for cancer treatment [32]. Combining siRNA with other anti-cancer drugs may offer a superior effect by enhancing therapeutic efficacy and overcoming drug resistance [33]. A previous study showed that sorafenib and siRNA of androgen receptors have additive effects to enhance sorafenib-induced apoptosis in prostate cancer [34]. The co-delivery of sorafenib and GPC3 siRNA has been shown to improve the sensitivity of HCC cells to sorafenib and increases the survival rate in a pre-clinical model [35]. In our study, a combination of TP53TG1 siRNA and sorafenib significantly inhibited cell growth and migration when compared to TP53TG1 siRNA or sorafenib when given alone. An additive effect of TP53TG1 siRNA plus sorafenib revealed the advantage of a combination therapy approach. Most notably, our data indicate that a lower dose of sorafenib can be used with TP53TG1 knockdown to achieve a maximal response, thus reducing potential toxicity.

In summary, our work indicates that the suppression of lncRNA TP53TG1 increases the anti-cancer effectiveness of sorafenib in HCC, indicating that targeting lncRNAs may be employed as part of a combination therapy to treat cancer.

## 4. Materials and Methods

### 4.1. Cell Culture and Transfection

The human HCC cell lines HepG2, PLC/PRF/5, and Hep3B were purchased from the Cell Culture Facility at Fox Chase Cancer Center. The HepG2 cell line was maintained in DMEM (Thermo Fisher Scientific, Waltham, MA, USA) supplemented with 10% cosmic calf serum (Hyclone Laboratories Inc., Logan, UT, USA) and 1% penicillin-streptomycin (Thermo Fisher). The PLC/PRF/5 cell line was grown in RPMI-1640 (Thermo Fisher) supplemented with 10% cosmic calf serum and 1% penicillin-streptomycin. The Hep3B cell line was cultured in DMEM/F12 (Thermo Fisher) supplemented with 10% cosmic calf serum and 1% penicillin-streptomycin. All cell lines were incubated at 37 °C in a 5% CO_2_ atmosphere.

Lacz small interfering RNA (siRNA) and TP53TG1 siRNAs were obtained from Sigma-Aldrich (St. Louis, MO, USA). Cell transfection was performed using Lipofectamine RNAiMAX (Thermo Fisher) following the manufacturer’s protocols. Si-TP53TG1 is a pool of two siRNAs (si-TP53TG1-1 and si-TP53TG1-2) that were used in our previous study [21]. The oligonucleotide sequences are: si-TP53TG1-1-F, 5′-CAAACUGUUUGGAAAGCUA-3′; si-TP53TG1-1-R, 5′-UAGCUUUCCAAACAGUUUG-3′; si-TP53TG1-2-F, 5′-CUUCCCUCUUAAUGAAUAA-3′; si-TP53TG1-2-R, 5′-UUAUUCAUUAAGAGGGAAG-3′; si-lacz-F, 5′-CUACACAAAUCAGCGAUUU-3′; si-lacz-R, 5′-AAAUCGCUGAUUUGUGUAG-3′.

### 4.2. Cell Counting kit-8 (CCK-8) Assay

Cell viability was determined by CCK-8 (Dojindo, Kumamoto, Japan) assay. Briefly, a total of 1.0 × 10^4^ HepG2 or 7 × 10^3^ PLC/PRF/5 cells transfected with si-lacz or si-TP53TG1 were seeded into each well of a 96-well plate and treated with or without sorafenib (Sigma, Burlington, MA, USA). A total of 10 µL of CCK-8 solution was added to each well, and the absorbance of each well was measured after 2 h of incubation by SpectraMax i3 at 450 nm.

### 4.3. RNA Extraction, Reverse Transcription, and qRT-PCR

Total RNA was extracted from HepG2, PLC/PRF/5, or Hep3B cell lines using TRIzol reagent (Thermo Fisher) and reverse transcribed into cDNA by a RevertAid RT Reverse Transcription kit (Thermo Fisher). A quantitative real-time polymerase chain reaction (qRT-PCR) was performed using LiQuant Universal Green qPCR Master Mix (LifeSct, Rockville, MD, USA). Primers were purchased from Integrated DNA Technologies, Inc. (TP53TG1-F, 5′-ACGAAGGTACCCAACCCTCT-3′; TP53TG1-R, 5′-TGTTCTTTTGCCAAGACACG-3′; 18S-F, 5′-AGTCCCTGCCCTTTGTACACA-3′; 18S-R, 5′-CGATCCGAGGGCCTCACTA-3′). The expression levels of RNA were normalized to 18S and determined using the 2^−ΔΔCt^ method.

### 4.4. Western Blotting

Proteins were extracted from HepG2 or PLC/PRF/5 cells using RIPA reagent (Cell Signaling Technology, Danvers, MA, USA) supplemented with protease and phosphatase inhibitors (Thermo Fisher). Proteins were resolved by SDS-PAGE gradient gel (Thermo) and transferred to a PVDF membrane (Sigma). Membranes were incubated overnight with the following primary antibodies: anti-ERK (1:1000, CST), anti-p-ERK (1:1000, CST), and anti-tubulin (1:1000, CST). Following washing, membranes were incubated with fluorescence conjugated secondary antibodies (LI-COR). The immunoreactive bands were visualized by a quantitative fluorescence imaging system (LI-COR).

### 4.5. Wound-Healing Assay

The migrations of HepG2 and PLC/PRF/5 cells were determined by wound-healing assay. Cells were plated into twelve-well plates and transfected. When they reached 100% confluence, the cells were wounded by a 200 μL pipet tip. Following this, the cells were washed with PBS and incubated with culture medium without serum. Images were taken using a microscope at 24 or 48 h after the initial scratch.

### 4.6. Statistical Analysis

All data are presented as mean ± standard error of the mean (SEM) unless specified otherwise. A one-way analysis of variance (ANOVA) or Students’ *t*-test was used for group comparison. A *p* value of <0.05 was considered statistically significant.

## Data Availability

Not applicable.
